# VEGF-A isoform-specific regulation of calcium ion flux, transcriptional activation and endothelial cell migration

**DOI:** 10.1242/bio.201410884

**Published:** 2015-04-24

**Authors:** Gareth W. Fearnley, Alexander F. Bruns, Stephen B. Wheatcroft, Sreenivasan Ponnambalam

**Affiliations:** 1 Endothelial Cell Biology Unit, School of Molecular & Cellular Biology, LIGHT Laboratories, University of Leeds, Leeds LS2 9JT, UK; 2 Division of Cardiovascular & Diabetes Research, Faculty of Medicine & Health, LIGHT Laboratories, University of Leeds, Leeds LS2 9JT, UK

**Keywords:** VEGF-A, NFATc2, Calcium, Endothelial, Cell migration

## Abstract

Vascular endothelial growth factor A (VEGF-A) regulates many aspects of vascular physiology such as cell migration, proliferation, tubulogenesis and cell-cell interactions. Numerous isoforms of VEGF-A exist but their physiological significance is unclear. Here we evaluated two different VEGF-A isoforms and discovered differential regulation of cytosolic calcium ion flux, transcription factor localisation and endothelial cell response. Analysis of VEGF-A isoform-specific stimulation of VEGFR2-dependent signal transduction revealed differential capabilities for isoform activation of multiple signal transduction pathways. VEGF-A_165_ treatment promoted increased phospholipase Cγ1 phosphorylation, which was proportional to the subsequent rise in cytosolic calcium ions, in comparison to cells treated with VEGF-A_121_. A major consequence of this VEGF-A isoform-specific calcium ion flux in endothelial cells is differential dephosphorylation and subsequent nuclear translocation of the transcription factor NFATc2. Using reverse genetics, we discovered that NFATc2 is functionally required for VEGF-A-stimulated endothelial cell migration but not tubulogenesis. This work presents a new mechanism for understanding how VEGF-A isoforms program complex cellular outputs by converting signal transduction pathways into transcription factor redistribution to the nucleus, as well as defining a novel role for NFATc2 in regulating the endothelial cell response.

## INTRODUCTION

There are 58 human receptor tyrosine kinases (RTKs) which are sub-classified into 20 families. These Type I membrane proteins regulate animal development, health and disease states (Lemmon and Schlessinger, 2010). Upon ligand-binding, RTK monomers undergo dimerisation followed by trans-autophosphorylation of cytoplasmic tyrosine residues, enabling the recruitment and phosphorylation of a vast array of signal transduction enzymes and adaptor proteins. RTKs are key targets for new therapeutics but successful drug design is complicated by the increasing number of ligands discovered to bind to each RTK. The physiological relevance for the expression of numerous ligands is unclear; many studies have based their conclusions on studying the effects of a single ligand for a specific RTK.

This complexity is exemplified by the human vascular endothelial growth factor (VEGF) family, which comprises 5 family members [VEGF-A, VEGF-B, VEGF-C, VEGF-D and placental growth factor (PlGF)]. Together this family of growth factors regulate angiogenesis and lymphangiogenesis through differentially binding to an array of Class V RTKs (VEGFR1-3) and co-receptors such as neuropilins i.e. NRP1 and NRP2 (Koch et al., 2011). The *VEGFA* gene is located on chromosome 6p21.3 (Vincenti et al., 1996); transcription of this gene leads to the formation of a pre-mRNA transcript with a coding region that contains 8 exons and 7 introns. Alternative splicing of the *VEGFA* mRNA transcript gives rise to at least 7 pro-angiogenic isoforms, which all bind to both VEGFR1 and VEGFR2 (Robinson and Stringer, 2001). However, it is also believed that, the pre-mRNA splicing machinery can also generate anti-angiogenic isoforms via alternate splice site selection events (Harper and Bates, 2008). These events termed proximal splice site selection (PSS) and distal splice site selection (DSS), determine the terminal amino acid sequence (exon 8) switching between the pro-angiogenic sequence CDKPRR (exon 8a) or the anti-angiogenic sequence SLTRKD (exon 8b) (Harper and Bates, 2008). This raises the question as to the functional relevance of the different VEGF-A isoforms; most studies have focused solely on the VEGF-A_165_ isoform, which is secreted by both vascular and non-vascular cells.

VEGF-A is a crucial regulator of angiogenesis, modulating diverse endothelial responses such as cell proliferation, migration, tubulogenesis, vascular permeability and leukocyte recruitment. *VEGFA* gene dosage is critical for normal development as heterozygous *VEGFA* (+/−) knockout mice embryos are not viable and die between E11 and E12 due to a deformed vascular network (Carmeliet et al., 1996; Ferrara et al., 1996). VEGFR1 and VEGFR2 can both bind different VEGF-A isoforms but it is unclear as to how the different RTK-ligand complexes regulate endothelial and vascular function. Nonetheless, both *VEGFR1* and *VEGFR2* encode gene products that are essential for correct vascular development and animal function (Fong et al., 1995; Shalaby et al., 1995).

VEGF-A binding to VEGFR2 triggers receptor dimerisation, linked to the activation of its tyrosine kinase domain, which triggers sustained downstream signal transduction integrated with receptor ubiquitination, trafficking and proteolysis (Bruns et al., 2009; Horowitz and Seerapu, 2012; Koch and Claesson-Welsh, 2012; Nakayama and Berger, 2013). A key aspect of VEGF-A-stimulated endothelial cell signal transduction is the elevated transcription of 100–200 target genes, which regulate a variety of cellular responses (Rivera et al., 2011; Schweighofer et al., 2009). Various studies have shown that VEGF-A isoforms differentially promote VEGFR2-dependent signal transduction and cellular outcomes (Kawamura et al., 2008a; Kawamura et al., 2008b; Zhang et al., 2000). However, the mechanism(s) which link VEGF-A isoform-specific signal transduction to nuclear gene transcription and endothelial responses are ill-defined.

To address the individual role of each VEGF-A splice isoform in regulating vascular function, we evaluated VEGF-A_121_ and VEGF-A_165_ for their ability to regulate signal transduction events linked to physiological responses. Here, we show that these two VEGF-A isoforms produce different intracellular signalling outcomes which impact on a transcriptional ‘switch’ allowing for isoform-specific regulation of endothelial cell migration. Thus, VEGF-A isoforms could act as temporal and spatial cues that program endothelial responses essential for building unique vascular networks.

## RESULTS

### VEGF-A isoforms cause differential VEGFR2 activation and signal transduction

**Fig. 1.**
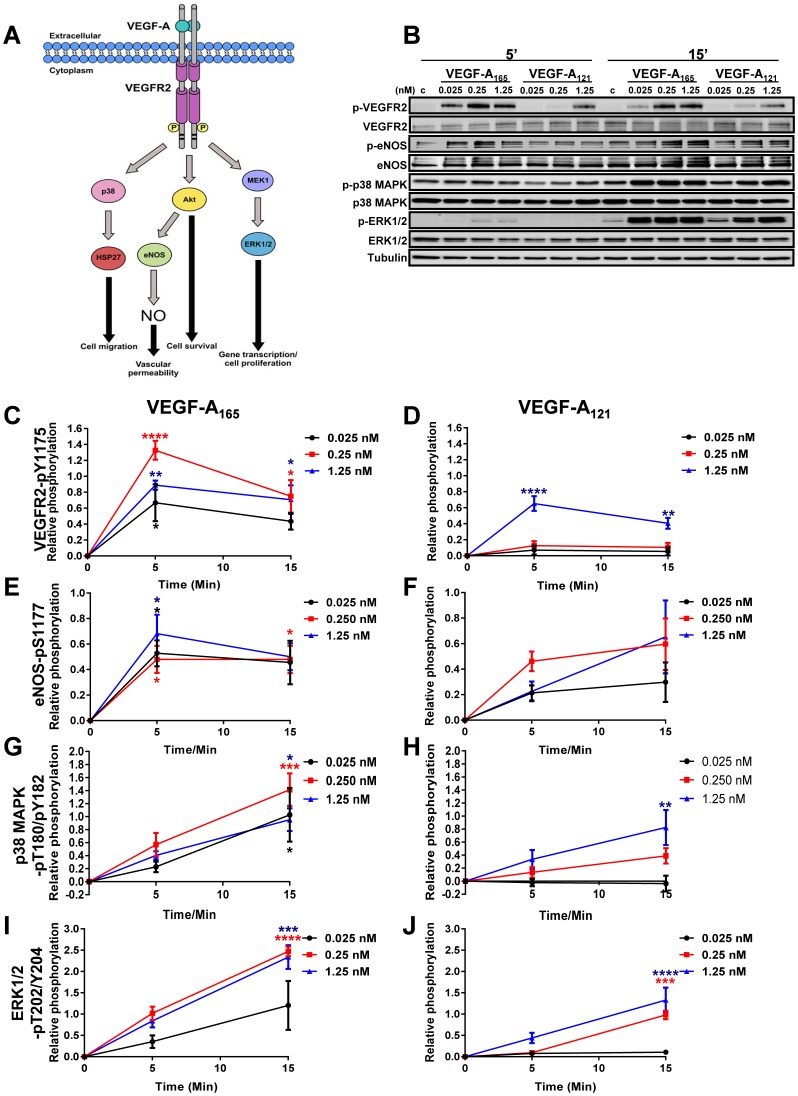
VEGF-A isoform-specific stimulated endothelial cell signalling. (A) Schematic depicting VEGF-A stimulated VEGFR2 phosphorylation, activation of downstream signalling pathways linking to cellular responses. Abbreviations: p38, p38 mitogen-activated protein kinase; HSP27, heat-shock protein of 27 kDa; Akt, Protein kinase B; eNOS, endothelial nitric oxide synthase; NO, nitric oxide; MEK1, mitogen-activated protein kinase 1; ERK1/2, p42/44 mitogen-activated protein kinase. (B) Endothelial cells subjected to different VEGF-A_165_ or VEGF-A_121_ concentrations (0, 0.025, 0.25 or 1.25 nM) for 5 or 15 min were lysed and processed for immunoblot analysis using phospho-specific antibodies against p-VEGFR2, p-eNOS, p-p38 and p-ERK1/2. (C–J) Quantification of VEGF-A isoform-specific signal transduction events. Quantification of (C,D) VEGFR2-pY1175, (E,F) eNOS-pS1177, (G,H) p38 MAPK-pT180/pY182 or (I,J) ERK1/2-pT202/Y204 upon (C,E,G,I) VEGF-A_165_ or (D,F,H,J) VEGF-A_121_ stimulation. Error bars indicate ±SEM (n≥3). *p*<0.05 (*), *p*<0.01 (**), *p*<0.001 (***), *p*<0.0001 (****).

VEGF-A-stimulation promotes VEGFR2 dimerisation and trans-autophosphorylation of several key tyrosine residues within the cytoplasmic domain (Koch and Claesson-Welsh, 2012) which stimulates downstream signal transduction pathways (Fig. 1A). Recruitment of factors and enzymes that bind activated VEGFR2 stimulates intracellular signalling events which modulate an array of endothelial cell responses in order to promote angiogenesis and regulate vascular development (Fig. 1A). Various studies have shown that VEGF-A isoforms promote differential VEGFR2 activation and downstream signal transduction (Kawamura et al., 2008b; Pan et al., 2007a). Although, VEGF-A-stimulated VEGFR2-dependent signalling is well understood, it is still unclear how VEGF-A isoform-specific signal transduction is converted into nuclear gene transcription to differentially regulate endothelial cell responses. In order to further investigate this phenomenon, we first compared the ability of two VEGF-A isoforms (VEGF-A_165_ and VEGF-A_121_) to regulate signal transduction events via the VEGFR2/VEGF-A signalling axis. Primary human umbilical vein endothelial cells (HUVECs) were titrated with 0.025, 0.25 and 1.25 nM of either VEGF-A_165_ or VEGF-A_121_ for 5 or 15 min prior to processing and immunoblot analysis of VEGFR2 activation and downstream signalling pathways (Fig. 1B). Quantification of the relative changes in phosphorylation status of VEGFR2-pY1175 in response to a dose-dependent titration of VEGF-A_165_ (Fig. 1C) or VEGF-A_121_ (Fig. 1D) revealed that peak activation occurred within 5 min of ligand treatment. However, VEGF-A_121_-stimulated VEGFR2-Y1175 phosphorylation (Fig. 1D) was significantly reduced versus VEGF-A_165_ treatment (Fig. 1C) at 0.025 and 0.25 nM. However, upon stimulation with saturating levels of VEGF-A (1.25 nM), the peak level of VEGFR2 activation achieved in response to VEGF-A_165_ (Fig. 1C) was comparable to that induced by VEGF-A_121_ (Fig. 1D). Interestingly, at this VEGF-A concentration, activated VEGFR2 appeared to be dephosphorylated at an increased rate upon treatment with VEGF-A_121_ (Fig. 1D) compared to cells treated with VEGF-A_165_ (Fig. 1C).

Angiogenesis is a tightly regulated process and many endothelial cell responses constantly need fine-tuning for homeostasis and maintenance of vascular physiology (Carmeliet, 2005). VEGF-A stimulation regulates several endothelial cell responses through the activation of downstream signalling events including those that affect endothelial cell permeability (Akt-eNOS pathway), cell migration (p38 MAPK pathway) and cell proliferation (ERK1/2 pathway). Stimulation with either VEGF-A_165_ or VEGF-A_121_ promoted phosphorylation of endothelial nitric oxide synthase (eNOS), p38 MAPK and ERK1/2 (Fig. 1B) enzymes. Quantification of eNOS-pS1177 levels revealed that stimulation with VEGF-A_121_ (Fig. 1F) resulted in a generally lower level of activation compared to VEGF-A_165_ stimulation (Fig. 1E). However, peak levels of eNOS activation were comparable between the two isoforms (Fig. 1E,F). Additionally both VEGF-A isoforms caused a similar increase in p38 MAPK-pT180/pY182 levels at 1.25 nM ligand stimulation, with peak levels detected after 15 min (Fig. 1G,H). In contrast VEGF-A_165_ promoted ∼2-fold increase in ERK1/2-T202/Y204 phosphorylation compared to VEGF-A_121_ at the same concentration (1.25 nM); peak levels were still detected 15 min post stimulation (Fig. 1I,J). These findings suggest that VEGF-A isoforms have the capability to differentially stimulate multiple signal transduction pathways in endothelial cells.

### VEGF-A isoforms promote differential PLCγ1 activation and corresponding cytosolic calcium ion flux

**Fig. 2.**
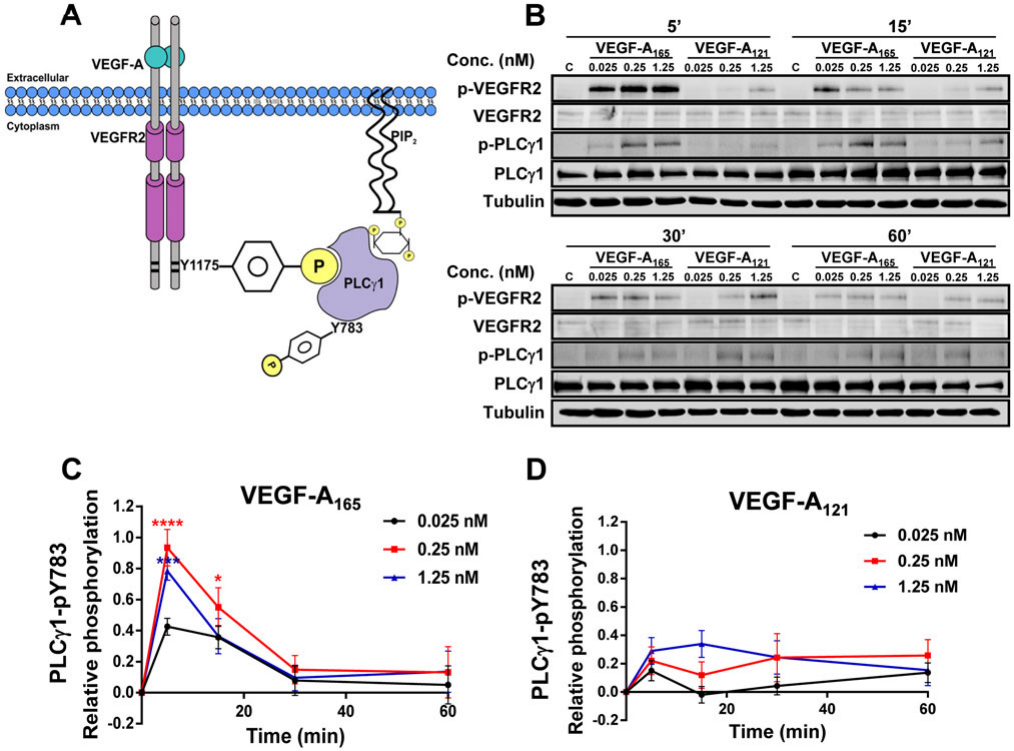
VEGF-A isoform-specific stimulated PLCγ1 activation. (A) Schematic depicting VEGF-A-stimulated VEGFR2-pY1175 recruitment of PLCγ1, subsequent phosphorylation and activation. (B) Endothelial cells subjected to different VEGF-A_165_ or VEGF-A_121_ concentrations (0, 0.025, 0.25 or 1.25 nM) for 5, 15, 30 or 60 min were lysed and probed for phospho-PLCγ1. (C,D) Quantification of PLCγ1-pY783 levels upon (C) VEGF-A_165_ or (D) VEGF-A_121_ stimulation. Error bars indicate ±SEM (n≥3). *p*<0.05 (*), *p*<0.001 (***), *p*<0.0001 (****).

VEGF-A-stimulated generation of VEGFR2-pY1175 creates a phosphotyrosine-based epitope that recruits PLCγ1 to activated VEGFR2 at the plasma membrane (Takahashi et al., 2001). Subsequently, VEGFR2-mediated PLCγ1 phosphorylation on residue Y783 (PLCγ1-pY783) leads to enzymatic activation and hydrolysis of plasma membrane PIP_2_ to DAG and InsP_3_ thus triggering a rise in cytosolic calcium ion flux and altered endothelial responses (Fig. 2A). To investigate VEGF-A isoform-specific PLCγ1-mediated responses, we monitored PLCγ1-pY783 levels upon titration with VEGF-A_165_ or VEGF-A_121_ for 5, 15, 30 or 60 min (Fig. 2B). PLCγ1-pY783 levels were clearly elevated upon addition of VEGF-A_165_ compared to VEGF-A_121_ (Fig. 2B) addition to endothelial cells. Quantification revealed VEGF-A_165_ stimulated a rapid and transient rise in endothelial PLCγ1-pY783 levels within 5 min (Fig. 2C) but VEGF-A_121_-stimulated PLCγ1-pY783 peak levels were ∼2–3-fold lower (Fig. 2D).

**Fig. 3.**
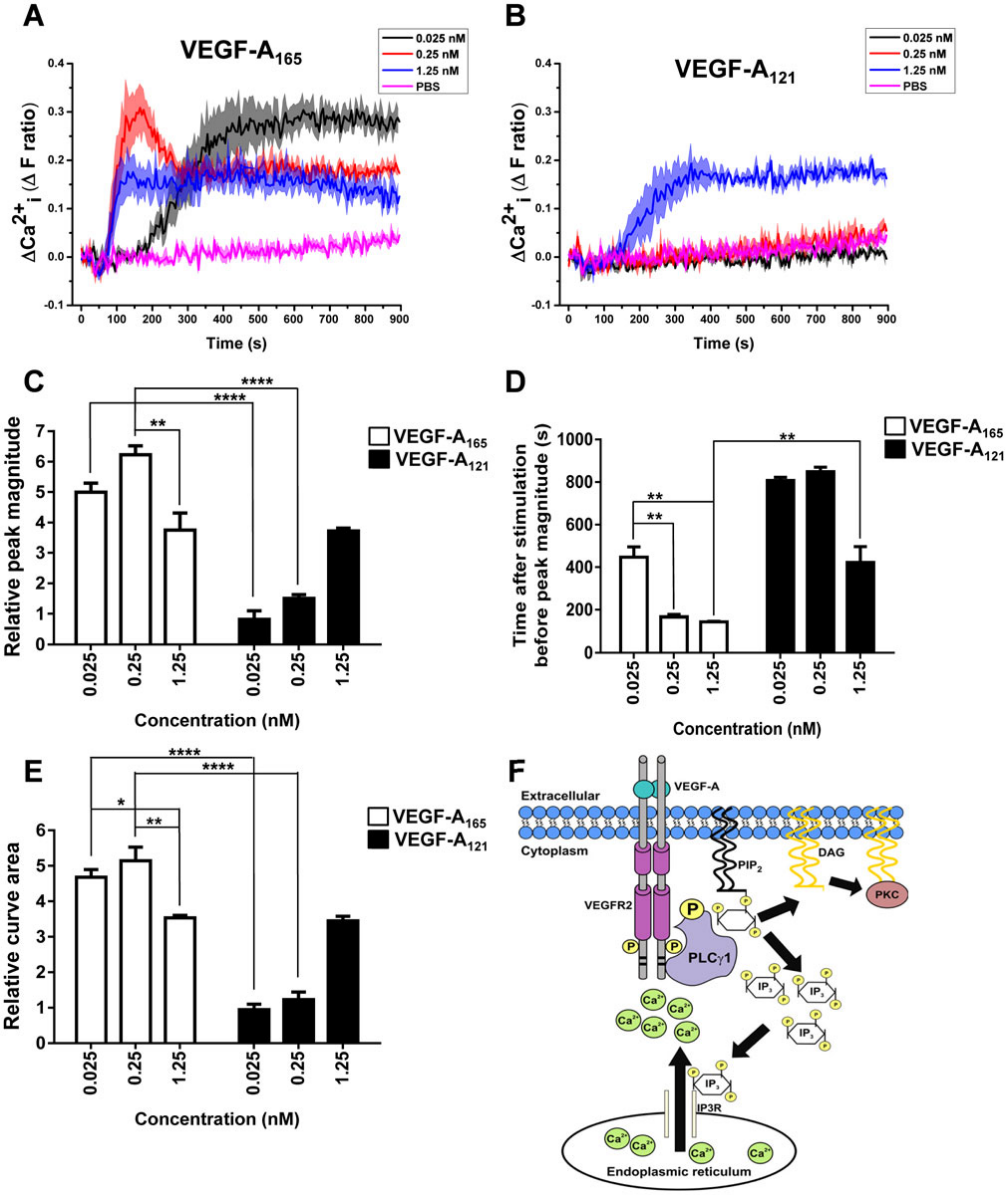
**VEGF-A isoform-specific release of intracellular Ca**^**2+**^**.** (A,B) Real-time monitoring of cytosolic Ca^2+^ flux with (A) VEGF-A_165_ or (B) VEGF-A_121_ titration; graphs show a representative plot of multiple experiments. Error bars indicate ±SEM (n = 1, N = 4). (C–E) Quantification of (C) peak magnitude, (D) time taken to reach peak magnitude or (E) the total curve area upon stimulation with VEGF-A_165_ and VEGF-A_121_. Error bars indicate ±SEM (n = 3). (F) Schematic depicting VEGF-A-stimulated second messenger targeting of InsP_3_ receptors (InsP_3_R) and subsequent cytosolic calcium ion rise. PtdIns(4,5)P_2_ hydrolysis to generate diacylglycerol (DAG) and Ins(1,4,5)P_3_ is depicted. *p*<0.05 (*), *p*<0.01 (**), *p*<0.0001 (****).

We then carried out an analysis of cytosolic calcium ion levels in VEGF-A isoform-stimulated endothelial cells using a cell-permeable Ca^2+^-sensitive fluorescent probe (Fura-2 AM). Upon titration of either VEGF-A_165_ (Fig. 3A) or VEGF-_A121_ (Fig. 3B) we observed different patterns of cytosolic calcium ion flux. The lowest concentration (0.025 nM) of VEGF-A_165_ ligand caused a slow and sustained rise in cytosolic calcium ions (Fig. 3A); this was not observed upon treatment with 0.025 nM VEGF-A_121_. A similar trend was observed at 0.25 nM ligand concentration of both isoforms (Fig. 3A,B). Interestingly, the highest concentration (1.25 nM) of ligand elicited similar rises in cytosolic calcium ions with both VEGF-A_165_ (Fig. 3A) and VEGF-_A121_ (Fig. 3B). Quantification of peak magnitude in ligand-stimulated rise in cytosolic calcium ion levels revealed that both VEGF-A_165_ and VEGF-A_121_ acted in a concentration-dependent manner (Fig. 3C). VEGF-A_165_ stimulation generally promoted an increased level in intracellular Ca^2+^ versus treatment with VEGF-A_121_ (Fig. 3C). However, at saturating levels of VEGF-A (1.25 nM) the peak magnitude of cytosolic calcium ion rise was comparable upon stimulation with either VEGF-A_165_ or VEGF-A_121_ (Fig. 3C). In contrast, the time taken to reach this peak magnitude was ∼2-fold longer in VEGF-A_121_-stimulated cells in comparison to VEGF-A_165_ treatment (Fig. 3D). Quantification of the relative curve area at different VEGF-A concentrations revealed a similar magnitude of response for VEGF-A_165_ regardless of concentration; however, VEGF-A_121_ only induced significant changes in intracellular calcium ion levels at the maximum concentration of 1.25 nM (Fig. 3E). These findings suggest that the cytosolic calcium ion flux caused by the VEGFR2-PLCγ1 axis is VEGF-A isoform-dependent (Fig. 3F).

### VEGF-A isoform-dependent nuclear and membrane protein localisation

**Fig. 4.**
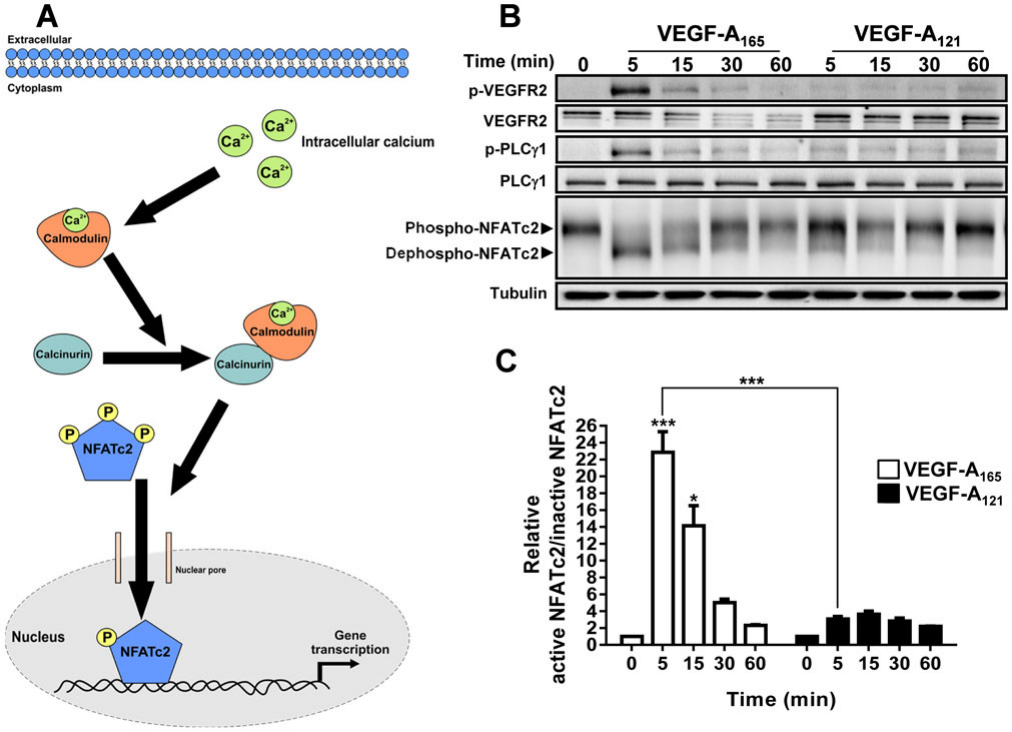
**VEGF-A isoform-specific Ca**^**2+**^ **flux promotes differential NFATc2 activation.** (A) Schematic depicting VEGF-A-stimulated cytosolic calcium ion rise and effects on NFATc2 dephosphorylation and nuclear translocation. (B) Endothelial cells subjected to stimulation with 0.25 nM VEGF-A_165_ or VEGF-A_121_ for 5, 15, 30 and 60 min were lysed and process for immunoblot analysis to detect phospho- (inactive) and dephospho- (active) NFATc2 species. (C) Quantification of relative active versus inactive NFATc2 levels upon stimulation with VEGF-A_165_ and VEGF-A_121_. Error bars indicate ±SEM (n = 3). *p*<0.05 (*), *p*<0.001 (***).

Cytosolic calcium ion fluxes can regulate endothelial cell gene transcription via the calcium-regulated and calmodulin-dependent activation of the protein phosphatase calcineurin (Domínguez-Rodríguez et al., 2012; Zarain-Herzberg et al., 2011). This enzyme regulates dephosphorylation and subsequent nuclear translocation of the nuclear factor of activated T-cells (NFAT) family of transcription factors (Rao et al., 1997; Wu et al., 2007) (Fig. 4A). VEGF-A can promote nuclear translocation of NFAT family members such as NFATc2 (Goyal et al., 2012; Zaichuk et al., 2004). We hypothesised that the differential VEGF-A isoform-specific regulation of cytosolic calcium ion flux could modulate activation of endothelial NFATc2. To test this idea, endothelial cells were stimulated with 0.25 nM of either VEGF-A_165_ or VEGF-A_121_ which elicited the major differences in cytosolic calcium ion flux, followed by immunoblot analysis of NFATc2 phosphorylation status (Fig. 4B). This revealed that at the peak of VEGF-A_165_-stimulated PLCγ1 phosphorylation (PLCγ1-pY783), there was a corresponding increase in NFATc2 activation, reflecting dephosphorylation of this transcription factor (Fig. 4B). However, at a comparable level of VEGF-A_121_, we found a relatively small rise in NFATc2 activation (Fig. 4B). Quantification of the relative levels of dephosphorylated (active) vs. phosphorylated (inactive) NFATc2 revealed that both VEGF-A isoforms simulated rapid and transient dephosphorylation, with peak dephospho-NFATc2 levels occurring after ∼5 min (Fig. 4C). However, VEGF-A_165_ stimulation promoted ∼22-fold increase in dephospho-NFATc2, whereas VEGF-A_121_ stimulation caused a significantly lower rise (∼2-fold) in dephospho-NFATc2 levels (Fig. 4C).

**Fig. 5.**
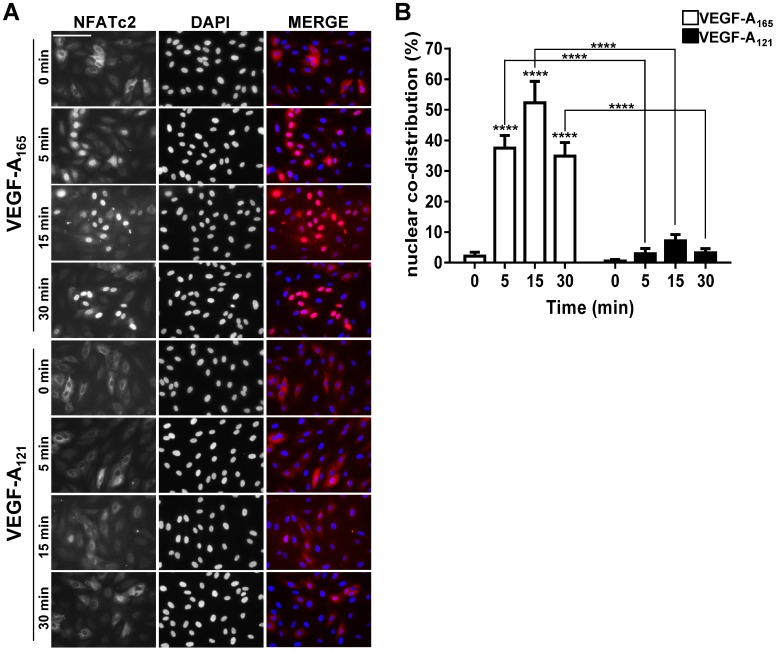
VEGF-A isoforms promote differential NFATc2 nuclear translocation. (A) Endothelial cells stimulated with 0.25 nM VEGF-A_165_ or VEGF-A_121_ for 5, 15 and 30 min were fixed and processed for immunofluorescence microscopy using rabbit anti-NFATc2 (green); nuclei stained using DAPI (blue). Scale bar, 1000 µm. (B) Quantification of NFATc2 nuclear co-distribution at 0, 5, 15 and 30 min after stimulation with VEGF-A_165_ or VEGF-A_121_. Error bars indicate ±SEM (n = 3). *p*<0.0001 (****).

Nuclear translocation of dephosphorylated NFATc2 is required for the modulation of endothelial gene expression (Wu et al., 2007). To test the idea that VEGF-A isoform-specific NFATc2 dephosphorylation regulates its nuclear translocation we evaluated the intracellular distribution of NFATc2 in response to VEGF-A isoform stimulation (Fig. 5). Endothelial cells were stimulated with either VEGF-A_165_ or VEGF-A_121_ (0.25 nM) for 0, 5, 15 or 30 min before fixation and processing for immunofluorescence analysis (Fig. 5A). Addition of VEGF-A_165_ caused rapid and sustained NFATc2 accumulation within the nucleus within a 30 min period (Fig. 5A). However, VEGF-A_121_ stimulation resulted in relatively low nuclear accumulation within a 30 min period (Fig. 5A). Quantification of NFATc2 nuclear co-distribution in a ligand- and time-dependent manner revealed ∼18-fold (VEGF-A_165_) or ∼3-fold (VEGF-A_121_) increase in peak nuclear NFATc2 levels (Fig. 5B). Interestingly, biochemical analysis of NFATc2 phosphorylation status after VEGF-A_165_ stimulation for 30 min showed that phosphorylated activated NFATc2 levels had returned to baseline (Fig. 4B) but the morphological analysis revealed a major pool of NFATc2 still present within the nucleus at the 30 min time point (Fig. 5A,B). Thus initial dephosphorylation of NFATc2 is sufficient to promote rapid cytosol-to-nuclear translocation but nuclear retention occurs via a different mechanism.

Cytosolic calcium ion fluxes have also been linked to promoting trafficking of another endothelial receptor tyrosine kinase (VEGFR1) from the Golgi apparatus to the plasma membrane (Bruns et al., 2009; Mittar et al., 2009). Due to the different abilities of the two VEGF-A isoforms in promoting the release of intracellular calcium ion stores (Fig. 3A–E), we hypothesised about effects on VEGFR1 translocation to the plasma membrane. To test this idea, we stimulated endothelial cells with either VEGF-A_165_ or VEGF-A_121_ for 0, 15, 30 or 60 min prior to assessing plasma membrane VEGFR1 using cell surface biotinylation (supplementary material Fig. S1A). Immunoblot analysis of mature and soluble VEGFR1 expression revealed that in non-stimulated endothelial cells, both forms of VEGFR1 are predominately located within internal, biotin probe-inaccessible compartments and not at the plasma membrane (supplementary material Fig. S1A). However, upon stimulation with VEGF-A_165_ there was a significant increase (∼2.5-fold) in both mature (supplementary material Fig. S1B) and soluble (supplementary material Fig. S1C) VEGFR1 (sVEGFR1/sFlt1) at the cell surface. Contrastingly, VEGF-A_121_ treatment failed to promote a significant increase in cell surface levels of either mature or sVEGFR1 (supplementary material Fig. S1A,B).

### VEGF-A-stimulated endothelial cell migration is NFATc2-dependent

**Fig. 6.**
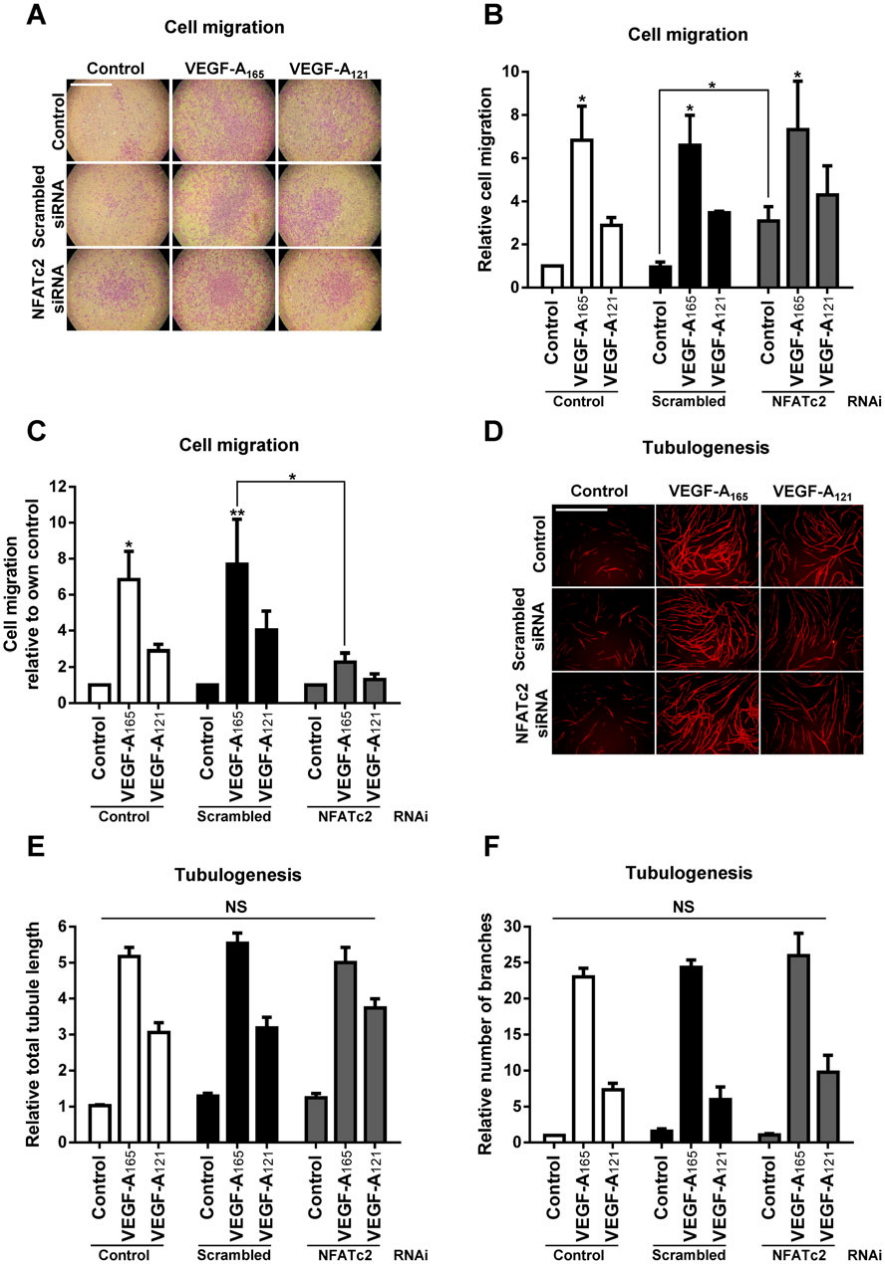
NFATc2 regulates VEGF-A isoform-specific endothelial cell migration but not tubulogenesis. (A–F) Control, scrambled or NFATc2-specific siRNA duplex-treated endothelial cells were seeded into assays to assess endothelial cell (A–C) migration or (D–F) tubulogenesis. (A) Endothelial cells seeded into Transwell filters were stimulated with 0.25 nM VEGF-A_165_ or VEGF-A_121_ for 24 h before being fixed and stained with 20% (v/v) crystal violet. Scale bar, 1000 µm. (B,C) Quantification of endothelial cell migration compared to (B) non-transfected or (C) individual controls. Error bars indicate ±SEM (n≥3). (D) Endothelial cells subjected to different siRNA treatments were co-cultured on a bed of primary human fibroblast for 7 days and stimulated with 0.25 nM VEGF-A_165_ or VEGF-A_121_. Co-cultures were fixed and endothelial tubules were stained and visualised using an anti-PECAM1 antibody followed by fluorescent secondary antibody. Scale bar, 1000 µm. (E,F) Quantification of endothelial cell tubulogenesis including total (E) tubule length or (F) number of branch points. Error bars indicate ±SEM (n≥3). *p*<0.05 (*), *p*<0.01 (**), NS = non-specific.

VEGF-A-stimulated signal transduction regulates a diverse number of long-term endothelial cells responses, such as cell migration and tubulogenesis (Chung and Ferrara, 2011; Koch et al., 2011). VEGF-A isoforms have been shown to differentially regulate endothelial cell responses (Kawamura et al., 2008b). However, the transcription factors involved in regulating these isoform-specific cellular responses are not well defined. To determine whether NFATc2 is required for the diverse array of VEGF-A-stimulated endothelial cell responses, we used reverse genetics combined with ligand-stimulated cellular assays (Fig. 6). We used siRNA duplexes to knockdown NFATc2 levels in endothelial cells and compared to scrambled siRNA duplex-treated or non-transfected controls (Fig. 6). Treatment with NFATc2-specific siRNA duplexes caused ∼90% knockdown in endothelial NFATc2 levels (supplementary material Fig. S2A,B), but did not affect expression of other endothelial proteins such as VEGFR2, VEGFR1 and ERK1/2 (supplementary material Fig. S2A). To determine the effect of NFATc2 knockdown on endothelial cell migration and tubulogenesis, NFATc2-depleted or control endothelial cells were stimulated with 0.25 nM VEGF-A_165_ or VEGF-A_121_, before fixation, staining and processing for light microscopy (Fig. 6A). As previously reported elsewhere, VEGF-A_165_ has a ∼2–3-fold higher efficacy for promoting endothelial cell migration (Fig. 6B,C). Interestingly, quantification revealed that depletion of NFATc2 caused ∼2–3-fold increase in basal cell migration in non-stimulated endothelial cells (Fig. 6B), during comparison of all values to non-transfected non-stimulated endothelial cells. In such an analysis, there appears to be relatively little change in VEGF-A isoform-stimulated cell migration upon scrambled siRNA treatment or NFATc2 knockdown (Fig. 6B). However, when each experiment was compared to their own non-stimulated control which had been also subjected to scrambled or NFATc2 siRNA, a different pattern emerged (Fig. 6C). There was now a substantial 3–4-fold decrease in both VEGF-A_165_ and VEGF-A_121_-stimulated endothelial cell migration in NFATc2 depleted endothelial cells (Fig. 6C).

An important aspect of the VEGF-A-stimulated endothelial cell response is the capacity to build hollow tubes (tubulogenesis) which can be monitored using an organotypic assay. Using this technique, we asked whether NFATc2 was required for VEGF-A-stimulated tubulogenesis (Fig. 6D). As previously reported elsewhere, VEGF-A_165_ has a ∼2–3-fold higher efficacy for promoting endothelial cell tubulogenesis, in comparison to VEGF-A_121_ (Fig. 6E,F). Intriguingly, NFATc2 did not significantly affect VEGF-A stimulated endothelial cell tubulogenesis (Fig. 6D). Quantification revealed that NFATc2 knockdown did not affect endothelial tubule length (Fig. 6E) nor branch point complexity (Fig. 6F).

## DISCUSSION

**Fig. 7.**
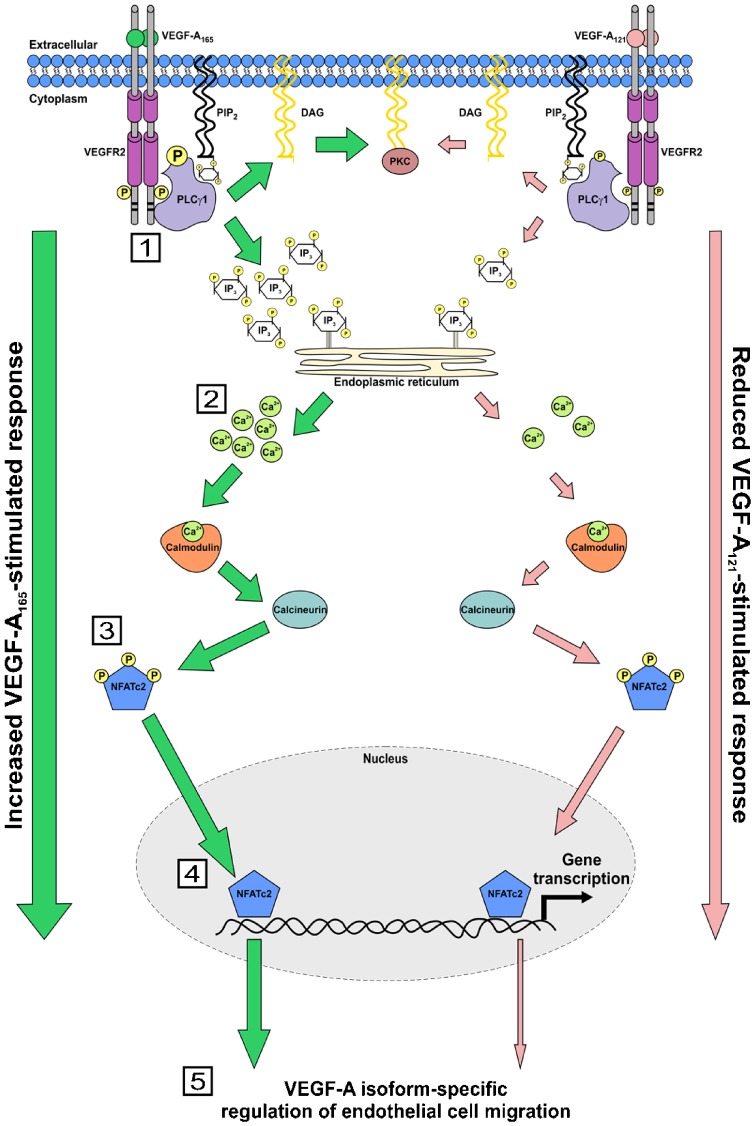
Mechanism for VEGF-A isoform-specific regulation of endothelial cell migration. Schematic depicting VEGF-A isoform-specific regulation of NFATc2 activation and regulation of endothelial cell migration. Numbered steps denote: (1) VEGF-A isoforms promote differential recruitment and activation of PLCγ1 through interaction with VEGFR2; (2) due to increased PLCγ1 activation, VEGF-A_165_ promotes an increased rise in intracellular Ca^2+^, versus VEGF-A_121_; (3) Increased VEGF-A_165_ stimulated intracellular Ca^2+^ flux results in increased NFATc2 dephosphorylation, versus VEGF-A_121_; (4) dephosphorylated NFATc2 translocates into the nucleus where it regulates endothelial gene transcription; (5) VEGF-A-stimulated NFATc2 regulated gene expression promotes endothelial cell migration.

In this study, we show that different VEGF-A isoforms have differing capabilities to modulate endothelial cell migration by regulating the activation and nuclear localisation of a key transcription factor (Fig. 7). In our proposed model, two VEGF-A isoforms with similar binding affinities differentially program VEGFR2 activation and downstream signal transduction which controls a transcriptional ‘switch’ that regulates endothelial cell migration. This ‘switch’ comprises the calcium-dependent activation of the calmodulin-calcineurin pathway which targets endothelial NFATc2, a key transcription factor (Fig. 7).

A key feature of endothelial cell migration is the VEGF-A isoform-specific programming of signal transduction and this cellular response. VEGF-A_165_ significantly promoted increased VEGFR2 phosphorylation at residue Y1175 in comparison to VEGF-A_121_. The generation of this unique pY1175 binding site enables recruitment of PLCγ1 via its SH2 domains and subsequent phosphorylation on residue Y783. VEGF-A isoform-specific stimulation of PLCγ1-pY783 levels correlated with increased cytosolic calcium ion flux (Fig. 7). Calmodulin is a key target of elevated cytosolic calcium ion levels and upon calcium ion binding to one of its four EF hands, undergoes a conformational change that promotes interaction with new cellular targets, including the protein phosphatase calcineurin (Berchtold and Villalobo, 2014). Calmodulin binding to calcineurin promotes enzymatic activation; one such target is NFATc2, resulting in its subsequent rapid dephosphorylation and nuclear targeting (Luo et al., 1996; Okamura et al., 2000). Both VEGF-A_165_ and VEGF-A_121_ stimulation promotes cytosolic calcium ion flux, NFATc2 dephosphorylation and nuclear translocation but to widely differing extents (Fig. 7). Depletion of endothelial NFATc2 levels significantly reduced VEGF-A-stimulated endothelial cell migration suggesting that this factor plays a role in controlling gene expression linked to cell migration processes e.g. focal adhesion, stress fibre formation and actin polymerisation (Fig. 7). Although, our results show that VEGF-A isoforms promote NFATc2 translocation into the nucleus, a lacking area of investigation is to determine if VEGF-A-stimulated NFATc2 dephosphorylation and nuclear translocation leads to the transcription of well-known NFATc2 regulated genes. However, it is highly likely that this is the case, due to the effect that depletion of NFATc2 has on VEGF-A-stimulated endothelial cell migration.

Various transcription factors such as ATF-2, NFAT, STAT3, forkhead-like transcription factors, FoxO- and ETS-related transcription factors are implicated in VEGF-A-dependent gene expression and physiological responses (Abid et al., 2006; Armesilla et al., 1999; Bartoli et al., 2003; Dejana et al., 2007; Fearnley et al., 2014a; Liu et al., 2003; Potente et al., 2005). However, the role of NFATc2 in VEGF-A stimulated angiogenesis is poorly defined. Previous studies have suggested a role for the transcriptional activity of endothelial NFATc2 in mediating VEGF-A-stimulated retinal angiogenesis (Zaichuk et al., 2004) and endothelial cell tubulogenesis (Goyal et al., 2012). Thus, our study now identifies a novel role for NFATc2 in the regulation of VEGF-A isoform-specific endothelial cell migration (Fig. 7). However, in contrast to work elsewhere (Bala et al., 2012), we did not find that NFATc2 was essential for VEGF-A-stimulated endothelial cell tubulogenesis. One possible explanation lies within the choice of assay used to monitor endothelial cell tubulogenesis. In our study we used a 7 day human fibroblast-endothelial cell co-culture assay to monitor the formation of PECAM-1 positive endothelial tubules, where Bala and colleagues performed a Matrigel co-culture assay (Bala et al., 2012); one drawback of this assay is that does not give an accurate index of endothelial cell tube formation (Donovan et al., 2001). Another reason could be the method used to look at NFATc2 involvement in endothelial cell function. In our study we used RNAi to directly deplete 90% of endothelial NFATc2. Bala and colleagues were studying the effects of a Kaposi's Sarcoma Herpesvirus protein (K15) on stimulating NFATc2-dependent RCAN1 expression (Bala et al., 2012). Therefore, it is possible that VEGF-A and K15-stimulated NFATc2 activation play different roles in determining endothelial cell fate. The fact that NFATc2 does not appear to be required for endothelial tube formation but is required for endothelial cell motility is surprising, as the latter is required for the former. This is a serious limitation to our current study and further work is required, the use of more robust 3-D angiogenesis assays such as the fibrin bead and ‘*ex vivo*’ mouse aortic ring assay would help strengthen the conclusions made here. Additionally, the monitoring of endothelial cell motility in real-time would further strengthen the role of NFATc2 in regulating VEGF-A isoform-specific cell migration. Likewise, analysis of other VEGF-A-stimulated endothelial responses (e.g. cell proliferation and permeability) for NFATc2 dependency would prove informative.

Interestingly, we found that basal NFATc2 activity inhibits the rate of cell migration in non-stimulated endothelial cells. One possible explanation could lie within the fact that NFATc2 naturally functions in an auto-inhibitory manner by promoting the expression of regulator of calcineurin 1 (RCAN1). RCAN1 forms a complex with calcineurin to directly inhibit its phosphatase activity, thus preventing calcineurin-dependent NFATc2 dephosphorylation and nuclear translocation (Holmes et al., 2010; Li et al., 2011). However, calcineurin also activates receptor for activated protein kinase C1 (RACK1), which has been implicated in regulating cell migration through binding to an array of signalling proteins. Endothelial cells constitutively express RCAN1 at a basal level (Holmes et al., 2010; Méndez-Barbero et al., 2013). Thus, depleting NFATc2 could reduce basal levels of RCAN1, subsequently leading to increased endothelial cell migration through attenuated calcineurin-stimulated RACK1 signalling. Calcineurin inhibitors have also been shown to inhibit cell migration (Espinosa et al., 2009; Siamakpour-Reihani et al., 2011), thus strengthening this proposed mechanism. RCAN1 is one of the most highly expressed gene products in response to VEGF-A-stimulation (Rivera et al., 2011; Schweighofer et al., 2009). Therefore, it is likely to have an essential role in regulating VEGF-A mediated endothelial responses. Various studies have shown that RCAN1 is essential for cellular migration (Espinosa et al., 2009; Holmes et al., 2010; Iizuka et al., 2004; Ryeom et al., 2008). Hence, VEGF-A isoform-specific elevation in RCAN1 expression could account for the reduction in VEGF-A-stimulated endothelial cell migration in NFATc2-depleted endothelial cells. Additionally, VEGF-A isoform-specific NFATc2-dependent upregulation of RCAN1 could also account for the differences in their abilities to stimulate endothelial cell migration.

One question left unanswered is what is the mechanism(s) behind how these two VEGF-A isoforms with similar binding affinities, promote such diverse activation of VEGFR2 and downstream signalling enzymes. One possible answer is through the differential binding and recruitment of co-receptors (e.g. NRP-1, NRP-2 and HSPG). Neuropilin 1 (NRP-1) is a VEGF-A isoform-specific co-receptor (Zachary et al., 2009). VEGF-A_165_ binds to the co-receptor neuropilin 1 (NRP1) to form a VEGFR2/NRP1/VEGF-A_165_ signalling complex (Fantin et al., 2013; Herzog et al., 2011; Raimondi and Ruhrberg, 2013; Zachary et al., 2009). Formation of this trimeric complex has been shown to increase VEGF-A_165_-stimulated VEGFR2 activation, downstream signalling and endothelial responses (Allain et al., 2012; Kawamura et al., 2008a; Koch, 2012). However, VEGF-A_121_ binding simultaneously to VEGFR2 and NRP1 has been contradicted (Pan et al., 2007b). Thus VEGF-A isoform-specific recruitment of NRP-1 could account for the differences in signalling between the 2 isoforms.

VEGF-A-stimulated NFATc2-dependent gene expression is said to occur in co-operation with the transcription factor early growth response 1 (EGR-1) (Schweighofer et al., 2007). VEGF-A stimulates EGR-1 gene expression through the MEK1-ERK1/2 pathway (Schweighofer et al., 2007). As we show that VEGF-A_165_ and VEGF-A_121_ have differential effects on ERK1/2 activation, one future study would be to investigate if these 2 isoforms differentially activated EGR-1 as a means of programing isoform-specific endothelial cell responses.

VEGFR1 binds VEGF-A with a much higher affinity than VEGFR2 (de Vries et al., 1992), yet its involvement in VEGF-A stimulated angiogenesis is not well understood. One view is that both membrane-bound and sVEGFR1 proteins act primarily as VEGF-A ‘traps’, thus limiting ligand availability for the major pro-angiogenic receptor, VEGFR2 (Rahimi, 2006). However, VEGF-A-regulated VEGFR1-linked signal transduction has been implicated in certain aspects of endothelial cell physiology (Koch and Claesson-Welsh, 2012). In non-stimulated endothelial cells, VEGFR1 is primarily inaccessible to VEGF-A, as it is located within an internal compartment resembling the Golgi apparatus (Mittar et al., 2009). VEGF-A_165_ stimulation promotes VEGFR1 translocation to the cell surface via a cytosolic calcium ion-dependent mechanism where it can bind exogenous VEGF-A. In this study, VEGF-A_165_ promoted significant trafficking of VEGFR1 to the plasma membrane whereas VEGF-A_121_ was largely ineffective in this context; again this could be explained by signal transduction effects on cytosolic calcium ion levels. The role of VEGF-A isoforms in differential VEGFR1 trafficking could further modulate the endothelial cell response to this ligand, as not only would this affect VEGF-A bioavailability for VEGFR2, but regulate VEGFR1-specific signal transduction in response to VEGF-A, VEGF-B and PlGF isoforms and thus regulate vascular physiology.

The physiological process of angiogenesis can be destabilised in a wide variety of major disease states ranging from atherosclerosis, rheumatoid arthritis, pathogenic infection to cancer. Current anti-angiogenic therapies which try to restrict pathological angiogenesis, by sequestering endogenous VEGF-A or via inhibiting RTK activity, are not as successful as first hoped. This is partially due to tumour cell-acquired resistance through the activation of alternative angiogenic pathways, in addition to other assorted mechanisms (Vasudev and Reynolds, 2014). Therefore, further investigation into the mechanisms which regulate angiogenesis is required. This will hopefully lead to improved patient outcomes in various disease states, through the design of better therapeutics. Our study now provides a novel mechanism to explain how different VEGF-A isoforms act on a common receptor tyrosine kinase (VEGFR2) to differentially determine a specific endothelial cell outcome (i.e. cell migration; Fig. 7). Additionally, this work shines new light on the physiological importance of NFATc2 in regulating VEGF-A-stimulated endothelial cell migration. A substantial future challenge will be to determine the biological significance of each VEGF-A isoform in healthy and diseased states.

## MATERIALS AND METHODS

### Antibodies and growth factors

Antibodies: goat-anti-VEGFR2 (R&D Systems, Minneapolis, MN, USA), goat-anti-VEGFR1 (R&D Systems), rabbit-anti-ERK1/2, mouse-anti-phospho-ERK1/2 (Thr^202^/Tyr^204^), rabbit-anti-p38, rabbit-anti-phospho-p38 (Thr^180^/Tyr^182^), rabbit-anti-phospho-VEGFR2 (Tyr^1175^), rabbit-anti-eNOS, rabbit-anti-phospho-eNOS(Ser^1177^), rabbit-anti-phospho-PLCγ1(Tyr^783^), rabbit-anti-NFATc2 (Cell Signaling Technology, Danvers, MA, USA), mouse anti-PECAM-1 (CD31), rabbit-anti-PLCγ1 (Santa Cruz Biotechnology, Dallas, TX, USA) and mouse-anti-α-tubulin (Sigma-Aldrich, Poole, UK). Reagents: Endothelial cell growth medium (ECGM) was from PromoCell (Heidelberg, Germany). Scrambled and NFATc2 siRNA duplexes were purchased as siGENOME SMARTpools from Dharmacon (Thermo Scientific, Lafayette, USA). Recombinant human VEGF-A_165_ was from Genentech Inc. (San Francisco, CA, USA) and VEGF-A_121_ was from Promocell.

### Cell culture

Human umbilical vein endothelial cells (HUVECs) were isolated as previously described (Howell et al., 2004). Human umbilical cords were obtained after written informed consent from volunteers. Tissue retrieval was governed by local ethics approval from the Leeds Hospitals NHS Trust. HUVECs were only used between passage P1 and P5. Cells were seeded into 6-well plates and cultured (for at least 24 h) in ECGM until ∼80% confluent, washed three times with PBS and then starved in MCDB131 + 0.2% (w/v) BSA for 2–3 h. HUVECs were then stimulated with 0, 0.025, 0.25 or 1.25 nM of VEGF-A_165_ or VEGF-A_121_ prior to processing.

### Cell lysis and processing for immunoblotting

Cells were washed with ice-cold PBS, prior to lysis in 2% (w/v) SDS in TBS containing 1 mM PMSF and protease inhibitor cocktail (Sigma-Aldrich) and stored at −20°C. Protein concentration was then determined using the bicinchoninic acid (BCA) assay (ThermoFisher). Protein lysate was diluted in an equal amount of 2× SDS-sample buffer and subjected to SDS-PAGE prior to analysis via immunoblotting.

### Cytosolic calcium ion flux assay

2.5×10^4^ HUVECs were seeded per well of a 96-well plate and cultured for 48 h until 100% confluent. Cells were washed twice with 100 µl SBS buffer (130 mM NaCl, 5 mM KCl, 1.2 mM MgCl_2_, 8 mM glucose, 10 mM HEPES, 1.5 mM CaCl_2_, pH 7.4) and loaded with 50 µl Fura-2 AM/SBS (2 µM Fura-2-AM, 0.01% Pluronic F-127; Life Technologies, Paisley, UK) for 60 min at 37°C. Cells were then washed twice with 100 µl SBS and left at room temperature for 30 min to allow complete de-esterification of Fura-2-AM. Desired concentration of VEGF-A isoform was made up as a 5× stock in a compound plate. Cytosolic calcium ion rise was monitored by measuring the ratio of 510 nm emission achieved from excitation at 340 nm vs. 380 nm using a FlexStation Benchtop Microplate Reader (Molecular Devices, Sunnyvale, USA). VEGF-A was added automatically after 32 s and Ca^2+^ levels were measured every 5 s for a total of 900 s. Change in cytosolic Ca^2+^ fluctuations as a function of time were plotted and quantified by calculating the peak magnitude, time take to reach peak magnitude and the area under the curve using OriginPro 8.6 (OriginLab, USA).

### Immunofluorescence analysis of NFATc2 localisation

HUVECs cultured in 96-well plates were serum starved for 3 h before being stimulated with 0.25 nM VEGF-A_165_ or VEGF-A_121_. Media was aspirated and cells incubated with fixative (Sigma-Aldrich) for 5 min at 37°C before permeabilisation in 0.2% (v/v) Triton X-100 in PBS. Cells were blocked in 5% (w/v) BSA prior to incubation with rabbit-anti-NFATc2 in 1% (w/v) BSA overnight at 4°C. Primary antibody was aspirated and cells washed, before incubation with 4 µg/ml donkey anti-rabbit Alexaflour488-conjugated secondary antibody (Invitrogen, Amsterdam, Netherlands), 2 µg/ml 4,6-diamidino-2-phenylidole (DAPI) in 1% (w/v) BSA in PBS and incubated for 2–3 h at room temperature. Images were acquired using an EVOS-fl inverted digital microscope (Life Technologies). 3 random fields were captured per sample. Relative nuclear co-localisation was quantified using Image J as previously described elsewhere (Bruns et al., 2010; Jopling et al., 2011).

### Lipid-based transfection of siRNA duplexes

HUVECs were reversed transfected using siRNA duplexes and Lipofectamine RNAiMAX (Life Technologies). Per well of a 6-well plate, 15 µl of 2 µM siRNA duplex was added to 481 µl of serum/antibiotic-free OptiMEM (Life Technologies) and allowed to react at room temperature for 5 min. 4 µl of Lipofectamine was then added to the siRNA duplex/OptiMEM mixture, inverted briefly and incubated at room temperature for 20 min. 2.5×10^5^ HUVECs in 1 ml of OptiMEM were seeded, followed by immediate dropwise addition of the siRNA/lipofectamine mixture. Cells were incubated at room temperature for 30 min before being returned to the incubator. After 6 h total incubation, transfection media was aspirated and replaced with ECGM. Cells were allowed to recover for 72 h prior to analysis of endothelial responses.

### Cell migration assay

3×10^4^ HUVECs were seeded in MCDB131 media containing 0.2% (w/v) BSA onto a 8 µm pore size Transwell filter [pre-coated with 0.1% (v/v) pig skin gelatin (PSG)] inserted into a 24-well plate (BD Biosciences, Oxford, UK). MCDB131+0.2% (w/v) BSA containing±0.25 nM VEGF-A isoform was added to the lower chambers to stimulate cell migration. Cells were allowed to migrate for 24 h prior to fixation and staining with 0.2% (w/v) crystal violet in 20% (v/v) methanol. Prior to fixation endothelial cells were visualised by phase contrast microscopy to ensure the number of adherent cells were comparable between Transwells. This ensures that differences seen in the number of migrating cells, was indeed caused by a defect in cell motility and not due to defective cell adhesion. Non-migrated cells were removed from the upper chamber with a moist cotton bud. Transwell filters were imaged and the number of migratory cells counted as previously described (Fearnley et al., 2014b).

### Tubulogenesis assay

Primary human foreskin fibroblasts (Promocell) were cultured in 48-well plates until confluent in complete DMEM containing 10% FCS, 1% non-essential amino acids and 1% sodium pyruvate (Life Technologies). 6500 HUVECs were then seeded onto the fibroblast monolayer in a 1:1 mixture of complete DMEM containing 10% (v/v) FCS, non-essential amino acids, sodium pyruvate and ECGM and left to adhere for 24 h. Culture media was then removed and replaced with fresh ECGM ±VEGF-A as desired; media was replaced every 2–3 days for 7 days. Co-cultures were then fixed and blocked prior to overnight incubation with 1 µg/ml mouse anti-human PECAM-1 (CD31; Santa Cruz Biotechnology) at room temperature. Co-cultures were washed three times with PBS before incubation with 1 µg/ml donkey anti-mouse Alexaflour594-conjugate (Invitrogen) for 2–3 h at room temperature. Endothelial tubules were then visualised by immunofluorescence microscopy using an EVOS-fl inverted digital microscope (Life Technologies). Three random fields were imaged per well. Both the number of branch points and the total tubule length was then quantified from each photographic field using the open source software AngioQuant (www.cs.tut.fi/sgn/csb/angioquant) and values averaged. A more detailed methodology is available elsewhere (Fearnley et al., 2014b).

### Statistical analysis

Statistical analysis was performed using one-way analysis of variance (ANOVA) followed by Tukey's post-hoc test or two-way ANOVA followed by Bonferroni multiple comparison test using GraphPad Prism software (La Jolla, CA, USA). Significant differences between control and test groups are denoted with *p* values less than 0.05 (*), 0.01 (**), 0.001 (***) and 0.0001 (****) indicated. Graphical error bars denote ±SEM (standard error of mean).

### List of abbreviations

AKT, Protein kinase B; ATF-2, activating transcription factor 2; DAG, diacylglycerol; eNOS, endothelial nitric oxide synthase; ERK1/2, p42/44; FAK focal adhesion kinase; HSP27, heat shock protein-27; InsP_3_, Inositol trisphosphate; MEK1, Mitogen-activated protein kinase kinase 1; NFAT, nuclear factor of activated T-cells; p38, p38 mitogen-activated protein kinases; PI3K, phosphatidylinositol 3-kinase; PIP_2_, Phosphatidylinositol 4,5-bisphosphate; PKC, protein kinase C; PLCγ1, phospholipase Cγ 1; RTK, receptor tyrosine kinase.

## Supplementary Material

Supplementary Material
