# Supplementary material for: VEGF-A isoform-specific regulation of calcium ion flux, transcriptional activation and endothelial cell migration
Source: Biol Open. 2015 Apr 24;4(6):731–42. doi: 10.1242/bio.201410884 (PMC4467193; doi:10.1242/bio.201410884)
Supplement: Supplementary Material [file supp_4_6_731__index.html]

VEGF-A isoform-specific regulation of calcium ion flux, transcriptional activation and endothelial cell migration — VEGF-A isoform-specific regulation of calcium ion flux, transcriptional activation and endothelial cell migration — Supplementary Material 

# VEGF-A isoform-specific regulation of calcium ion flux, transcriptional activation and endothelial cell migration

## bio.201410884 Supplementary Material

**Files in this Data Supplement:**

- Supplementary Material - Gareth W. Fearnley et al. doi: 10.1242/bio.201410884
