# Supplementary material for: VEGF-A isoform-specific regulation of calcium ion flux, transcriptional activation and endothelial cell migration
Source: Biol Open. 2015 Apr 24;4(6):731–42. doi: 10.1242/bio.201410884 (PMC4467193; doi:10.1242/bio.201410884)
Supplement: Supplementary Material [file supp_bio.201410884_bio.201410884-s1.pdf]

Supplementary Material
Gareth W. Fearnley et al. doi: 10.1242/bio.201410884

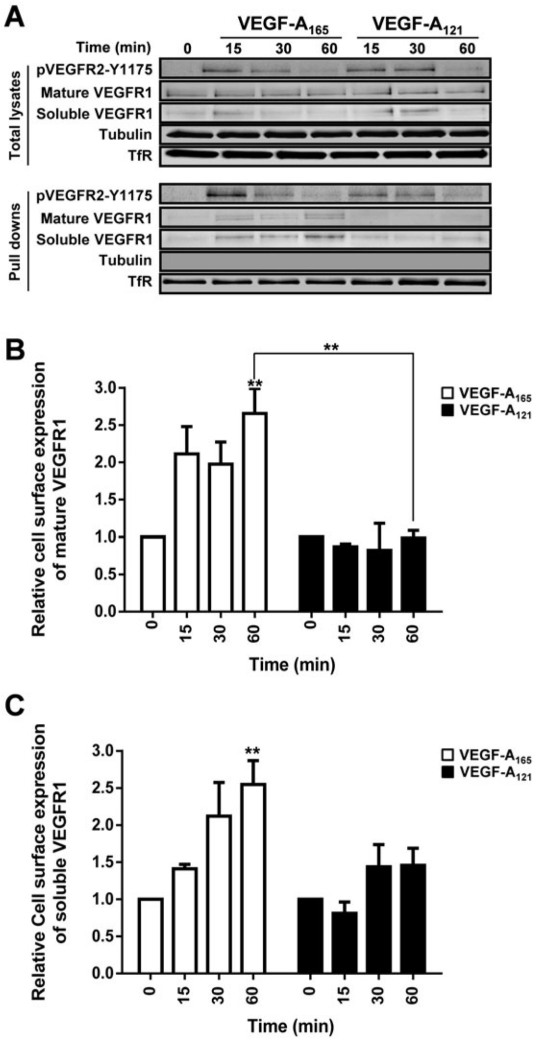

Fig. S1. VEGF-A isoforms promote differential trafficking of VEGFR1 intracellular pools. (A) Cell surface biotinylation, affinity isolation and immunoblot analysis of total cell lysates or biotinylated cell surface protein pool. Negative control (tubulin) and positive control (transferrin receptor, TfR). (B) Quantification of cell surface mature VEGFR1 or (C) soluble VEGFR1 levels upon VEGF-A_{165} or VEGF-A_{121} stimulation. Error bars indicate ±SEM (n=3). $p<0.01$ (**).

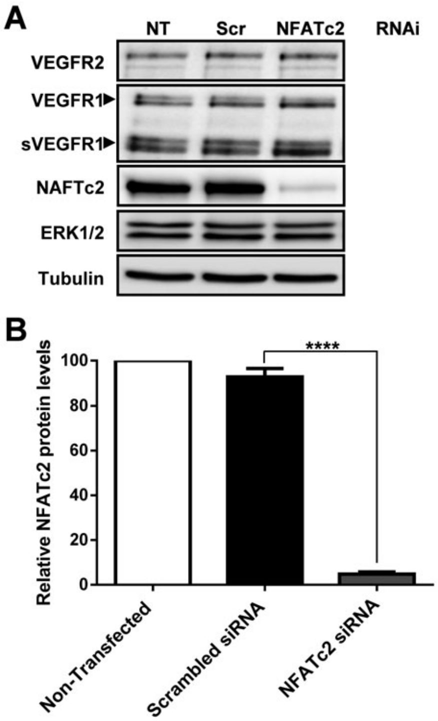

Fig. S2. Quantification of endothelial NFATc2 depletion upon treatment with specific-siRNA duplexes. (A) Control, scrambled or NFATc2-specific siRNA duplex-treated endothelial cells were lysed and processed for immunoblot analysis to determine NFATc2 protein expression levels. (B) Quantification of NFATc2 protein expression levels in control, scrambled or NFATc2-specific siRNA duplex-treated endothelial cells. Error bars indicate ±SEM (n=3). $p<0.0001$ (****).

Biology Open
